# Sputtering of Molybdenum as a Promising Back Electrode Candidate for Superstrate Structured Sb_2_S_3_ Solar Cells

**DOI:** 10.1002/advs.202303414

**Published:** 2023-09-05

**Authors:** Hu Li, Guo‐Qin Yang, Xiao‐Yang Hu, Yi‐Hua Hu, Rui‐Bo Zeng, Jin‐Rui Cai, Li‐Quan Yao, Li‐Mei Lin, Li‐Ping Cai, Guilin Chen

**Affiliations:** ^1^ Fujian Provincial Engineering Technology Research Center of Solar Energy Conversion and Energy Storage College of Physics and Energy Fujian Normal University Fuzhou 350117 China; ^2^ State Grid Dehua County Electric Power Supply Company Quanzhou 362500 China; ^3^ College of Computer and Cyber Security Fuzhou 350117 China

**Keywords:** molybdenum, Sb_2_S_3_ solar cells, Se layers, sputtering, superstrate

## Abstract

Sb_2_S_3_ is rapidly developed as light absorber material for solar cells due to its excellent photoelectric properties. However, the use of the organic hole transport layer of Spiro‐OMeTAD and gold (Au) in Sb_2_S_3_ solar cells imposes serious problems in stability and cost. In this work, low‐cost molybdenum (Mo) prepared by magnetron sputtering is demonstrated to serve as a back electrode in superstrate structured Sb_2_S_3_ solar cells for the first time. And a multifunctional layer of Se is inserted between Sb_2_S_3_/Mo interface by evaporation, which plays vital roles as: i) soft loading of high‐energy Mo particles with the help of cottonlike‐Se layer; ii) formation of surficial Sb_2_Se_3_ on Sb_2_S_3_ layer, and then reducing hole transportation barrier. To further alleviate the roll‐over effect, a pre‐selenide Mo target and consequentially form a MoSe_2_ is skillfully sputtered, which is expected to manipulate the band alignment and render an enhanced holes extraction. Impressively, the device with an optimized Mo electrode achieves an efficiency of 5.1%, which is one of the highest values among non‐noble metal electrode based Sb_2_S_3_ solar cells. This work sheds light on the potential development of low‐cost metal electrodes for superstrate Sb_2_S_3_ devices by carefully designing the back contact interface.

## Graphical abstract

Sb_2_S_3_ is regarded as one of candidate materials for tandem top cells. Here Mo as back electrode is employed in superstrate structured Sb_2_S_3_ solar cells for the first time, rendering a low cost and high stability. The mechanism of Mo deposition and device efficiency improvement are also explored in detail.

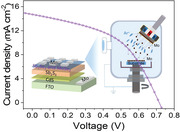

## 1 Introduction

Binary compound antimony sulfide (Sb_2_S_3_) has attracted a great deal of interest as a potential absorber candidate for photovoltaic applications due to its high absorption coefficient (1×10^5^ cm^–1^), abundant raw materials, and environment‐friendly characteristics.^[^ ^1^ ^]^ Moreover, its suitable band gap (≈1.7 eV) makes its theoretical efficiency up to ≈30%.^[^ ^2^ ^]^ However, there is a big gap between the recorded efficiency (8%) and the theoretical value.^[^ ^3^ ^]^ Comprehensive studies, such as device structures, film preparation, and interface engineering, have been well conducted. For most highly‐efficient Sb_2_S_3_ solar cells, they commonly use an organic hole transport layer (HTL) and/or noble Au electrode as the back contact materials (BCM). For example, the recorded power conversion efficiency (PCE) of Sb_2_S_3_ solar cells in dye‐sensitized and planar structures are 7.5% and 8% which are equipped with the PEDOT:PSS/PCPDTBT/Au and Spiro‐OMeTAD/Au, respectively.^[^ ^3^, ^4^ ^]^ Besides, the P3HT is also fully exploited by the Papadopoulos group and achieves a PCE of 3.97% of Sb_2_S_3_ solar cells.^[^ ^5^ ^]^ The outstanding physical property, i.e., suitable band level of these organic HTL, and high work function (*W*_F_, 5.1 eV) and conductivity (4.52×10^7^ S m^–1^) of Au, enables many groups to make great progress of Sb_2_S_3_ solar cells as shown in Table S1 (Supporting Information). However, the organic HTL and/or noble metal obviously limit the stability of the devices and also increase the manufacturing cost. Hence, it is urgent to develop a low‐cost and highly stable BCM for Sb_2_S_3_ solar cells. Encouraging, carbon has a W_F_ close to that of gold, which is also regarded as a candidate for low‐cost electrode materials to replace Au.^[^ ^6^ ^]^ But, the current carbon‐based Sb_2_S_3_ solar cells is still restricted by the high square resistance of carbon electrode, due to its porosity characteristics.^[^ ^7^ ^]^ Therefore, it is necessary to develop a cheap, stable, and highly conductive back electrode for Sb_2_S_3_ solar cells.

Mo, a kind of abundant metallic element (1.5 ppm) and lows‐cost (< 0.1% of that of Au), has attracted extensive attention in the field of optoelectronic devices and energy storage due to its excellent electrical conductivity (1.87×10^7^ S m^–1^) and high toughness.^[^ ^8^ ^]^ In particular, Mo shows a great application potential in the photovoltaic device by taking advantage of its high thermal stability and flexibility. For example, state‐of‐the‐art thin‐film solar cells, such as copper indium gallium selenide (CIGS) and copper zinc tin sulfoselenide (CZTSSe) routinely adopt Mo as a bottom electrode, then assembling into a substrate device.^[^ ^9^, ^10^ ^]^ Furthermore, Mo is also applied in substrate Sb_2_S_3_ thin film solar cells with Mo/Sb_2_S_3_/CdS/ZnO/AZO/Al structure, achieving a moderate efficiency of 3.75%.^[^ ^11^ ^]^ Here, we summary all the existing cases of Sb_2_S_3_ solar cells with Mo electrodes, demonstrating its infancy as shown in Table S2 (Supporting Information).^[^ ^12^, ^13^, ^14^, ^15^, ^16^, ^17^ ^]^ It is suggested that Mo is a potential electrode material for substrate Sb_2_S_3_ thin film solar cells, in which Mo is directly sputtered on an inert glass. This deposition process is harsh with high sputtering power and high temperature. Hence, to improve the buried interface of Mo and Sb_2_S_3_, modification of Mo electrode, including the regulation of its adhesion, conductivity, and surficial property, can be easily realized by experiencing the Mo electrode under high pressure, high‐temperature in situ deposition, and violent post‐sulfurization/selenization. However, up to now, Mo does not appear as the back electrode in the superstrate structured Sb_2_S_3_ solar cells, in which a simple architecture of TCO/ETL/Sb_2_S_3_/BCM is used (TCO: FTO, ITO; electron transport layer (ETL): TiO_2_, CdS, SnO_2_; BCM: Spiro‐OMeTAD and/or Au). The reason is that Mo metal possesses high thermodynamic stability and melting point, so it is inapplicable for the traditional chemical coating method or evaporation deposition route. By contrast, the magnetron sputtering strategy can perfectly deposit the Mo layer with a uniform morphology and is more suitable for industrial production. While the Sb_2_S_3_ film will suffer from the violent bombardment of high‐energy particles during the sputtering process and greatly deteriorate the surface structure of the Sb_2_S_3_ layer. The damaged surface inevitably introduces a mass of defect sites and then leads to severe recombination, which is a great challenge for superstrate structured Sb_2_S_3_ solar cells. This is the main consideration about the application of sputtering metal electrode for a superstrate solar cell and still pending for the non‐noble metal deposition. In addition, Mo will produce serious band alignment mismatches if it is directly combined with the Sb_2_S_3_ absorption layer, due to the low WF of Mo and deep valence band maximum (VBM) of Sb_2_S_3_. Therefore, how to solve the band‐level mismatch between Mo and absorption layer as well high‐energy Mo beam bombardment are vital to improve the PCE of low‐cost Sb_2_S_3_ solar cells.

In this work, we deposit Mo as the back electrode of a superstrate structured Sb_2_S_3_ solar cell by magnetron sputtering for the first time. And evaporation of a glue layer of Se is skillfully introduced between Sb_2_S_3_ and Mo electrodes to alleviate the high‐energy particle penetration during the sputtering process, namely the soft‐loading of Mo. The trade‐off between the shield function and carrier transport barrier of the Se layer is studied in detail by adjusting its thickness. In addition, an ultra‐thin Sb_2_Se_3_ buffer layer is detected on the Sb_2_S_3_ surface after the post‐annealing, attributing to trace residue of the Se shield layer, which logically up‐shifts VBM of Se‐modified Sb_2_S_3_ surface and then significantly improves hole collecting efficiency. However, due to the low *W*_F_ of the Mo electrode, small band bending hinders the hole transportation toward the Mo electrode, leading to a serious trailing state which results in an obvious cross‐over effect of the current density‐voltage (*J–V*) curve.^[^ ^18^ ^]^ Hence, we skillfully sputter a pre‐selenide Mo target which is aimed to insert a MoSe_2_ buffer layer between Sb_2_S_3_ and Mo. Hereafter, composite layers of MoSe_2_ and Mo are sputtered on the Sb_2_S_3_ film. This MoSe_2_ buffer layer effectively alleviates the energy level mismatch between Mo and Sb_2_S_3_ and then accelerates the transport of carriers and improves the efficiency of the device. Finally, a new device structure is assembled as FTO/CdS/Sb_2_S_3_/MoSe_2_/Mo and delivers a champion efficiency of 5.1% which is the highest PCE of non‐noble metal electrode‐based Sb_2_S_3_.

## 2 Results and Discussion

### 2.1 Superstrate Structured Sb_2_S_3_ Solar Cells with Mo Electrodes

**Figure 1:**
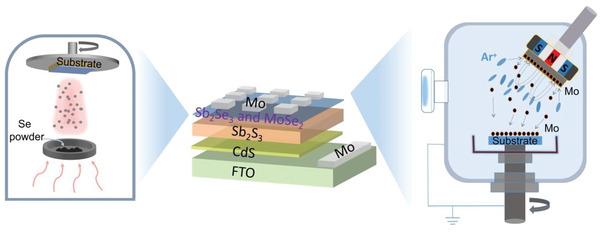
Schematic diagram of the back contact construction process for a superstrate Sb_2_S_3_ solar cell. Right sketch is the magnetron sputtering of Mo electrode, and left sketch is the thermal evaporation of Se layer.

Mo exhibits tremendous potential as electrodes in thin film solar cells by enjoying its high thermal stability, conductivity, and flexibility. It is often used as a bottom electrode either in CIGS, CZTSSe, or Sb_2_(S*_x_*,Se_1−_ *_x_*)_3_ (0≤*x*≤1) with substrate structures so far.^[^ ^19^, ^20^ ^]^ In this work, Mo is used as an electrode in superstrate structured Sb_2_S_3_ solar cells for the first time, displaying a simple device architecture of FTO/CdS/Sb_2_S_3_/Mo (**Figure** **1**). As a preliminary attempt, the Mo electrodes were deposited directly onto the as‐annealed FTO/CdS/Sb_2_S_3_ film by magnetron sputtering (150 °C, 100 W, 2 h), where the active area of 0.09 cm^2^ is defined by a mask. For more sputtering details, please refer to the Experimental Section.

**Figure 2:**
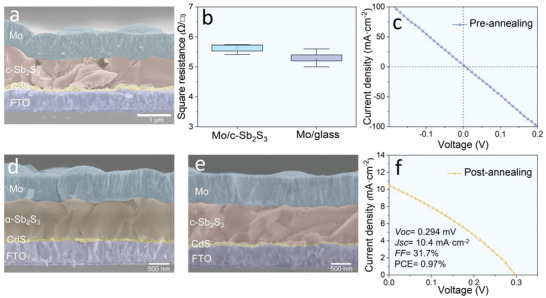
a) The cross‐sectional images of Mo deposited directly on c‐Sb_2_S_3_ films. b) Square resistance of Mo electrode deposited on c‐Sb_2_S_3_ layer or glass substrate. c) The *J–V* curve of corresponding Sb_2_S_3_ solar cell. The cross‐sectional images of Mo deposited on d) α‐Sb_2_S_3_ precursor films, and e) following a post‐annealing. f) The *J–V* curve of corresponding Sb_2_S_3_ solar cell with an integral post‐annealing.

First, we conducted a scanning electron microscopy (SEM) to explore the morphologies of the as‐prepared Sb_2_S_3_ films. It can be observed in Figure S1 (Supporting Information) that the annealed Sb_2_S_3_ films (crystalline phase, referred to as c‐Sb_2_S_3_) derived from the hydrothermal route exhibit a flat and uniform morphology which is the prerequisite for the subsequent sputtering deposition of Mo electrodes. As shown in **Figure** **2**a of the cross‐sectional SEM image, a high‐quality Mo electrode is directly deposited on the c‐Sb_2_S_3_ thin film by a facile magnetron sputtering, exhibiting a high uniformity and flatness attached with typical vertical columnar grains. It is well known that such a sputtering process is widely adopted to grow compact, uniform, tough, and highly conductive Mo electrodes, and displaying a similar columnar morphology when compared with the counterpart deposited on a glass substrate (Figure S2, Supporting Information). This is well consistent with Mo electrodes in substrate structured Sb_2_S_3_, as well as CIGS, CZTSSe solar cells.^[^ ^21^ ^]^ The conductivity, one of the most appealing characters of the metal electrodes, is further explored on devices by a four‐point probe test (Figure 2b). It is concluded that the square resistance of the Mo deposited on Sb_2_S_3_ surface (5.6 Ω □^–1^) is not distinctly different from that on the glass substrate (5.4 Ω □^–1^) in the same thickness of ≈900 nm under the standard sputtering process. Unexpectedly, the Mo‐attached Sb_2_S_3_ solar cells shows an awful device performance (Figure 2c), although the excellent characteristic of the Mo electrode is presented above. Almost no photoelectric response is observed in this case which may be caused by the high‐energy particles of Mo penetration to form shunting paths. In order to reveal the underlying reason, it can be seen from the topography of Figure 2a that Mo deposition is in good contact with the surface of c‐Sb_2_S_3_ layer, but can also be embedded at the grain boundary owing to the tiny holes. Based on this, we suggest that high energy particles of sputtered Mo particle would penetrate the c‐Sb_2_S_3_ layer through the grain boundary or the void created by grain fusion, and generating shunt paths. In order to mitigate the damage to the as‐prepared Sb_2_S_3_ film caused by the high energy particles, the sputtering power is reduced from 150 to 60 W. However, it still cannot avoid from device failure which can be seen in *J–V* curve of Figure S3 (Supporting Information). Hence, no devices are exempt from shunting once the Mo metal is directly sputtered on the as‐annealed Sb_2_S_3_ films.

As discussed above, the possible shunt path maybe created at grain boundary of the annealed Sb_2_S_3_ film. Hence seeking of a dense film gives the top priority to prevent the device breakdown against the energetic particles beam during Mo sputtering. As shown in Figure S4 (Supporting Information), we can find that the as‐prepared Sb_2_S_3_ precursor by hydrothermal method, exhibiting with amorphous property (referred to as α‐Sb_2_S_3_), is obviously denser than the c‐Sb_2_S_3_ polycrystalline film. Therefore, the Mo electrode is optimally deposited on α‐Sb_2_S_3_, which shows a tight contact with Sb_2_S_3_ precursor (Figure 2d). Sequentially, the samples are integrally annealed under N_2_ atmosphere to induce the grain growth of Sb_2_S_3_ and then consequentially reduce the bulk recombination. As displayed in Figure 2e, large grains are observed in c‐Sb_2_S_3_ film after post‐annealing, and the tight back contact interface is still reserved which is benefit for the effective carrier collection of Mo electrode. In this case, the Sb_2_S_3_ device performance has greatly improved from the initial resistance state to an obvious photoelectric response, which can be seen in Figure 2f. For example, the open circuit voltage (*V*_oc_) is boosted to 0.294 V, and current density (*J*_sc_) is enhanced to 10.4 mA·cm^−2^. But, the PCE of 0.97% is still poor, featured with relatively low *V*_oc_ and fill factor (*FF*), indicating the fatal destruction of high‐energy Mo particles cannot be completely avoided. Therefore, it is urgent to introduce a protective layer to solve the device shunting during sputtering Mo electrode.

### 2.2 Insertion of a Selenium Multifunctional Layer between Back Contact Interface

Selenium is an excellent semiconductor material, possessing charming features of stable single‐element component, high saturated vapor pressure (1.1 Pa @ 250 °C) and low melting point (220 °C), which is extensively used in the preparation and photoelectric property regulation of chalcogenide compounds.^[^ ^22^ ^]^ With regard to Sb_2_(S, Se)_3_ system, i) Sb_2_S_3_ and Sb_2_Se_3_ are isomorphic with same space group and similar cell parameters, indicating that S atom of Sb_2_S_3_ is easily replaced by Se atom. ii) It also bestows an excellent ability of bandgap engineering by facilely changing S/Se ratios. Then Sb_2_Se_3_ generated on the surface of Sb_2_S_3_ can facilitate the hole transport toward BCM by upshifting the deep VBM of Sb_2_S_3_. iii) Se layer can be used as a barrier layer to prevent the penetration of high‐energy particles, realizing soft landing of Mo during sputtering. iv) MoSe_2_ may be thermodynamically formed by reacting Se with Mo, and then effectively alleviates the energy level mismatch at back contact interface, which can accelerate the transport of carriers and improve the device PCE.

**Figure 3:**
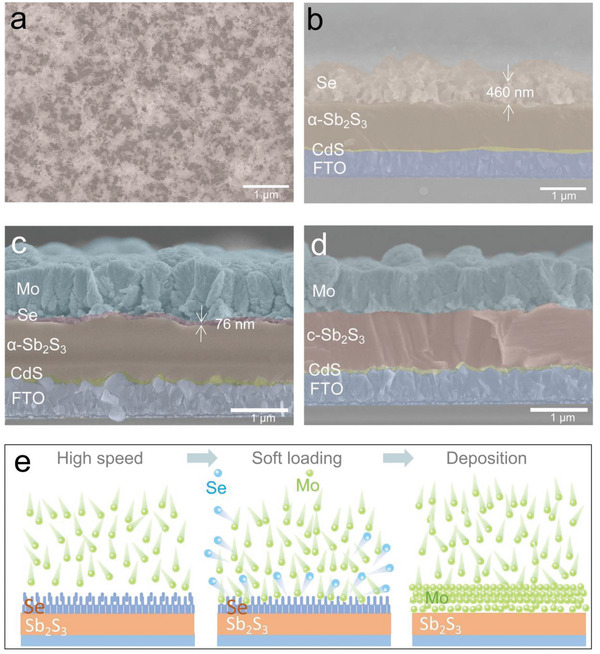
The a) surface and b) cross‐sectional images of 0.1 g Se coated Sb_2_S_3_ thin films. The cross‐sectional images of Sb_2_S_3_ thin films attached with 0.1 g Se layer and Mo electrode c) before and d) after annealing. e) The growth model of Mo electrode by sputtering with the assist of an additional Se layer.

In order to verify the above multi‐merits of Se, a glue Se layer is then employed by flash evaporation prior to the Mo electrode construction for Sb_2_S_3_ solar cells, please refer to left sketch of Figure 1. The as‐evaporated Se film on Sb_2_S_3_ surface shows a cotton‐like morphology with porous features and a thickness of 460 nm (**Figure** **3**a,b), which can be attributed by the low‐energy Se atoms during the flash evaporation. After sputtering of Mo electrodes, a Mo layer with rice grains is then coated on Sb_2_S_3_ precursor, also leaving a tiny porous selenium (≈76 nm) between them (Figure 3c). However, after annealing, a close connection of Mo and c‐Sb_2_S_3_ is observed in Figure 3d, in which the residual Se is consumed by reacting with Sb_2_S_3_ during post‐annealing (discussed in the following section). Such a resulted perfect back contact interface is benefit for the hole collection toward Mo electrodes. Here, we propose a sputtering growth model of Mo electrodes with the assist of an additional Se layer. As shown in Figure 3d, the sputtered Mo particle with high energy first approaches Sb_2_S_3_ surface and then collides with the cotton‐like Se layer, where this scatter‐shot will speed down the Mo particles and realize a soft loading of Mo without damage of Sb_2_S_3_. The similar energy absorption effect is observed in the porous materials, which also confirms that the as‐prepared porous selenium layer could effectively absorb the high energy of the sputtering Mo particles and serves as an important buffer layer. Concurrently, a large proportion of Se is scattered out from Se glue layer, leaving a trace residue of Se (Figure 3c). After complete coverage of the bottom layer of Mo, the upper Mo will then be deposited smoothly with a thickness of 800 nm within 2 h sputtering. This growth model is also confirmed by the structure of as‐deposited Mo electrode, as shown in Figure S10 (Supporting Information). The XRD result indicates the peak intensity of Mo at 2*θ* = 41.5° decreases significantly after adding 0.1 g Se prior to Mo deposition. This is due to fact that the Mo atoms with lower energy, as indicated in the second stage of growth model of Figure 3e, is not sufficient to migrate and induce metal recrystallization and finally degrade its crystallinity. Then the conductivity of as‐deposited Mo is tested by four‐point probe test in Figure S5 (Supporting Information) in consideration of the distinctive morphology and crystalline of Mo W/O or W/ selenium glue layer. Encouragingly, the Mo electrode coated with the help of Se layer still exhibits a relatively low square resistance of 6 Ω □^–1^ which is comparable with the counterpart of Mo on glass. In brief, the additional Se glue layers not only speed down the sputtered Mo atoms and form a benign contact interface, but also maintain an excellent conductivity property, which is essential for the vigorous sputtering of metal electrodes in superstate Sb_2_S_3_ solar cells.

**Figure 4:**
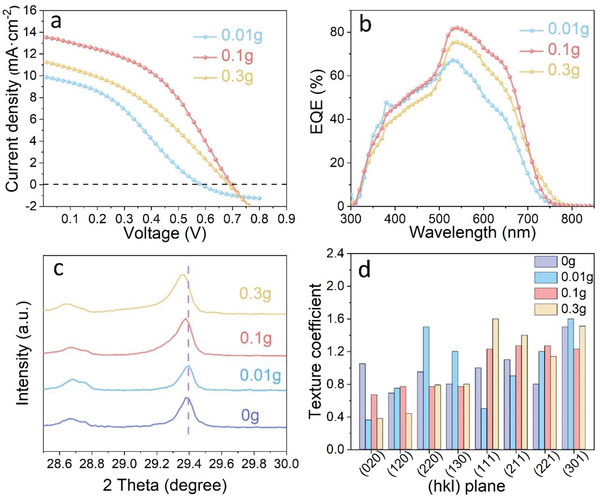
a) *J–V* and b) EQE curves of the Sb_2_S_3_ solar cell modified with 0.01, 0.1, and 0.3 g Se‐layer. c) Corresponding XRD patterns ≈2*θ* range 28.5–30.0° show a shift in the peak of (211). d) The texture coefficients (TC) deduced from XRD pattern.

To further reveal the effect of different thicknesses of Se layer on the quality of Mo back contact, various dosage of Se powder is used as 0.01, 0.1, and 0.3 g, labeled as 0.01–0.3 g Se‐layer hereinafter. Similar surface morphologies of Se layer are observed in both Figure 3a,b and Figure S6 (Supporting Information), exposing of a porosity trait. While different thicknesses of Se layers (190–800 nm) are detected from the cross‐sectional SEM images with feeding different dosage of Se powder source. To optimize the Se layer thickness, the *J–V* curves of the Sb_2_S_3_ solar cells with 0.01, 0.1, and 0.3 g Se‐layer were measured under simulated AM 1.5 (100 mW·cm^−2^) sunlight, as shown in **Figure** **4**a. More device metrics can be found in Table S3 (Supporting Information). The device with 0.01 g Se shows *J*_sc_ of 9.9 mA·cm^−2^, *V*_oc_ of 0.578 V, *FF* of 36.2%, and a PCE of 2.1%. The PCE of 0.01 g Se‐based device significantly outperforms that of 0g‐Se based device by 116%, especially for the improved *V*_oc_. It implies the Se layer can prevent the penetration by speeding down the Mo particles, as indicated in the above growth model. When the mass of Se increases to 0.1 g, the overall device metrics of the Sb_2_S_3_ solar cells are improved obviously, and delivering a PCE of 4.2%, along with *J*_sc_ of 13.6 mA·cm^−2^, *V*_oc_ of 0.699 V, and *FF* of 44.1%. However, further increasing the feeding dosage of Se (0.3 g) don't improve device performance, mainly reflecting in a significant reduction in *J*_sc_. To further confirm the device difference, as well as repeatability, the statistic boxplots of device parameters collected from 40 devices are exhibited in Figure S7 (Supporting Information). It is worth mentioning that 0.01 g Se devices have a large *V*_oc_ deviation, which is also related to the coverage of Se layer as discussed in the previous growth model. All device metrics, including *V*_oc_, *J*_sc_, *FF*, and PCE gain a remarkable improvement after employing a 0.01 g Se‐layer.

To disclose the function of Se layer with various thickness, the cross‐sectional morphology of device is revealed in Figure S8 (Supporting Information). As for 0.01 g Se‐layer case, the as‐sputtered Mo electrodes before or after annealing are all uniform and flat, featuring with a typical columnar grain, which is similar to the sample without a Se shield. Such a thin Se layer can alleviate the high‐energy Mo particles bombardment to a certain extent, but it still cannot be completely prevented, both of which leads to a moderate *V_oc_* of 0.577 V. With the increase of Se mass to 0.1 g, there is an amorphous Se layer ≈76 nm appeared between the Mo electrode and the absorption layer after sputtering Mo on it (Figure 3c), which is one sixth the as‐evaporation Se layer before sputtering. This again verifies the self‐sacrificing function of Se layer during the sputtering process, and endowing an appreciable soft‐loading deposition. And, after annealing (Figure 3d), the amorphous Se layer disappears and the back contact of the Sb_2_S_3_ device is obviously improved. The reaction of Se at back contact interface will discuss in the coming section. Once the Se mass increases to 0.3 g, however, a thick residual Se layer obviously appears between Sb_2_S_3_ and Mo electrode even after post‐annealing (Figure S8c,d, Supporting Information), which leads to a large gap between Sb_2_S_3_ layer and Mo electrode and then deteriorates the hole transportation toward Mo electrode. The resulted imperfect interface produces a large contact resistance, which pulls down both *J*_sc_ and *FF*. This is also verified by the external quantum efficiency (EQE) as shown in Figure 4b. It shows an inferior response of 0.01g‐based devices compared with the 0.1 g counterparts in the whole spectrum, owing to the short‐circuited channel captures the carriers. As for 0.3 g Se case, the response strength is also significantly lower than that of 0.1 g, because thick Se layer is not conducive to carrier transport between absorber and electrode due to the large gap as described above. In addition, a slight shift of absorption edge is observed when different dosage of Se is used. To clear it, the band gap of Sb_2_S_3_ film is determined by plotting the curve of EQE versus Energy. As shown in Figure S9 (Supporting Information), both 0.01 and 0.1 g Se‐based samples possess an almost identical band gap of ≈1.69 eV, which is close to that of untreated Sb_2_S_3_ film (0g‐Se). And the 0.1g‐Se case is then referred as Sb_2_S_3_ due to the same bandgap comparable with pristine Sb_2_S_3_. But, a massive dose of Se (0.3 g) observably narrows the band gap of Sb_2_S_3_ to 1.64 eV. So, the residual Se after sputtering of Mo should be kept in mind as it will sequentially react with Sb_2_S_3_ due to the low formation enthalpies of Sb_2_(S,Se)_3_ alloy.^[^ ^23^ ^]^

Here, the second role of Se will be studied, according to it also bestows an excellent regulation ability of crystalline structure and bandgap of Sb_2_S_3_ by facilely changing S/Se ratios. To verify this, the X‐ray diffraction (XRD) curves with respect to Se layer‐modified devices are exhibited in Figure S10 (Supporting Information). Clearly, all the diffraction peaks are well in line to the standard pattern of Sb_2_S_3_ (PDF#42‐1393) except the peaks for FTO and Mo substrate. And the Sb_2_S_3_ films show preferred orientation of (211). The close inspection found that the peak of (211) move to a low 2*θ* side with the increase of Se mass (Figure 4c), along with the change of intensity of each diffraction peak, which can be explained by the lattice expansion induced by the replacement of S^2−^ ion (1.84 Å) with larger Se^2−^ ion (1.98 Å). Then the resulted Sb_2_Se_3_ will improve the back contact interface by up shifting the deep VBM of pristine Sb_2_S_3_ surface. Deng et al. also observed the similar phenomenon that the addition of Sb_2_Se_3_ layers prepared by rapid thermal evaporation serves as a hole transport layers for superstrate Sb_2_S_3_ solar cells, which obviously reduces the back‐contact barrier and improve hole collection yield by enjoying the matched energy‐level alignments.^[^ ^2^ ^]^ On the other hand, in order to reveal the impact of post‐annealing on the orientation Sb_2_S_3_ thin films, the texture coefficient (TC) of the main (hk0) and (hk1) planes are calculated. As shown in Figure 4d, the TC value of the (hk1) in the Sb_2_S_3_ thin films increases after inserting 0.1g‐Se layers, while the (hk0) shows a opposite trend. This can be explained by top‐down growth model induced by the as‐generated Sb_2_Se_3_ seed layer. In our previous work, we propose two growth model affecting orientation of Sb_2_S_3_ films, such as substrate‐induced (bottom‐up) and surface‐induced growth (also denote as top‐down growth).^[^ ^6^ ^]^ Here, we verify the results by comparing it to bottom‐up growth mode, as shown in Figure S11 (Supporting Information). It can be seen that although the same precursor is adopted and then the main composition is undoubtedly identical (Figure S11a, Supporting Information), the preferred orientation is distinctly different (Figure S11b, Supporting Information). Applied the top‐down growth model, the as‐prepared Mo electrode was heated in advance during annealing with the inversion method which leads the preferential formation of the seed layer of Sb_2_Se_3_ as validated in Figure S12 (Supporting Information). When the heat treatment time is as short as 10 s, a small amount of Sb_2_Se_3_ with preferred (221)‐oriented plane is formed, and the strength of Sb_2_S_3_ gradually becomes stronger as the annealing duration becomes longer. Hence, the vertical [Sb_4_Se_6_]*_n_* standing on the bare Sb_2_S_3_ layer functions as a seed layer which helps to induce the (hk1)‐preferred orientation of Sb_2_S_3_ films getting away from the bound of substrate. It is well known that the (hk0)‐oriented grains manifests (Sb_4_S_6_)*_n_* ribbons lay flat on the substrate, while the counterpart of (hk1)‐oriented grains stack more vertically on the substrate which is beneficial to carrier transport along the perpendicular direction. Hence, the alloyed Sb_2_S_3_ thin film with the favored (hk1) orientation will render a more effective separation of photo‐induced carrier in the corresponding device. In preliminary summary, the introduction of a multifunctional Se layer can kill two birds with one stone. It not only enables the soft‐loading of Mo electrode, but also forms an effective Sb_2_Se_3_ layer to boost carrier transport, which means that the sputtering of Mo electrode for superstrate structured Sb_2_S_3_ solar cells is feasible.

### 2.3 Skillful Design of MoSe_2_ Buffer Layer for Sb_2_S_3_ Solar Cells

Although Mo can be successfully deposited as electrode for superstrate structured Sb_2_S_3_ solar cells with the help of Se layer, the device performance is still unsatisfactory. For example, Figure 4a shows the *J–V* characteristic curve of the optimized Sb_2_S_3_ device with 0.1g‐Se layer, displaying an obvious roll‐over effect (current saturation at high forward bias) under illumination. It reflects a serious hole‐collection barrier at back contact in Mo‐attached superstrate Sb_2_S_3_ device.^[^ ^24^ ^]^ Hence, seeking or modification of BCM with a suitable *W*_F_ are of primary task to build a preferred back contact interface. With regard to substrate chalcogenide device, pre‐selenization or post‐selenizaton is always conducted to generate a MoSe_2_ buffer layer on the surface of Mo bottom electrode, which can significantly improve the interfacial energy band alignments.^[^ ^25^, ^26^ ^]^ For example, Li et al. introduce an additional selenization process, forming a thin MoSe_2_ layer on the Mo surface, which was found to effectively improve the bottom Mo electrode contact and alleviate the roll‐over effect of substrate Sb_2_Se_3_ thin film solar cells.^[^ ^27^ ^]^ Inspirited by it, we first examine if the additional Se layer would convert Mo into MoSe_2_ during the post‐annealing. Unfortunately, whether it's 0.01, 0.1, or 0.3 g fed, there is no MoSe_2_ peaks observed in the XRD pattern (Figure S10, Supporting Information). The original conception is that the residual Se multifunctional layer may not only form a surficial Sb_2_Se_3_, but also generate MoSe_2_ between the Sb_2_S_3_ and Mo. However, the limitation of the annealing temperature is fixed as low as 350 °C mainly considering of the low melting point of Sb_2_S_3_.^[^ ^28^ ^]^ Such a low temperature is not enough to form MoSe_2_, which is again confirmed by the absence of MoSe_2_ in control Mo film annealed under Se atmosphere at the same temperature (Figure S13, Supporting Information). So how to successfully synthesize MoSe_2_ and incorporate it into the Mo‐based Sb_2_S_3_ device is very significant for alleviating the roll‐over effect.

**Figure 5:**
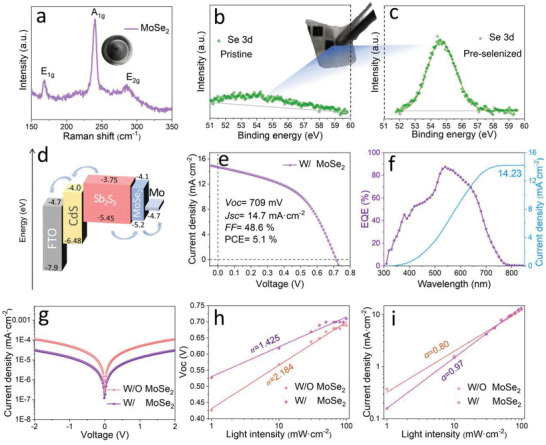
a) Raman spectra of the pre‐selenization Mo target. The XPS curves of the Mo electrode peeled by tape, which is sputtered from b) pristine c) pre‐selenized Mo target. d) The band alignment diagram of the Sb_2_S_3_ device. The e) *J–V* curve and f) EQE spectra of the champion Sb_2_S_3_ device with pre‐selenization Mo target. g) Dark *J–V* characteristics of the Sb_2_S_3_ thin‐film solar cells, and h) *V*_oc_ and i) *J*_sc_ as a function of light intensity.

Herein, we skillfully insert a MoSe_2_ buffer layer between Mo and Sb_2_S_3_ films. To simplify the sputtering procedure, a pre‐selenized Mo target is prepared by sequentially evaporating Se films on the surface of Mo target and then forming MoSe_2_ through post‐annealing at the high temperature of 500 °C for 20 min. As shown in **Figure** **5**a of Raman spectra of the pre‐selenized Mo target, the peaks located at 165 and 285 cm^−1^ corresponds to two weak vibration modes of *E*_1_g and *E*_2_g of MoSe_2_ while 242 cm^−1^ is assigned to the strong vibration mode of A_1_g, of MoSe_2_.^[^ ^29^ ^]^ These results confirm the formation of MoSe_2_ on the surface of Mo target. In order to uncover the sequential sputtering of MoSe_2_ and Mo, the pre‐selenzied target is sputtered on glass for 1 min under the same conditions as Mo target. The Raman spectra of as‐sputtered film only appears a main peak of MoSe_2_ at 242 cm^–1^ (Figure S14, Supporting Information) indicating the priority sputtering of surficial MoSe_2_. After sputtering with 10 min, the XPS results display the disappearance of Se element and detect only Mo peak (Figure S15, Supporting Information). It means that during sputtering, an ultra‐thin MoSe_2_ is first sputtered out followed by Mo electrode deposition. The electrode thickness with different deposition times was measured from cross‐sectional SEM and shown in Figure S16 (Supporting Information). It is obviously that the electrode thickness is almost linear with the sputtering duration, and the calculated deposition rate is ≈1.3 Å s^–1^. When the layer of MoSe_2_ (< 78 nm) is finished sputtering, it is followed by the deposition of the Mo electrode and thus the MoSe_2_/Mo composite is successfully constructed for superstrate Sb_2_S_3_ solar cells.

In order to clarify the back contact compound, we also use super glue (≥ 500 g/25 mm) to peel the back electrode of Mo from Sb_2_S_3_ surface for XPS testing. The XPS spectra of stripped electrode surface is recorded, and the Se 3*d* core levels is then shown in Figure 5b,c. It can be seen that the response of Se signal is not observed when the target doesn't experience pre‐selenization, whereas the pre‐treated target produces an intense response of Se peak, in which the peak located at binding energies of 54.50 is ascribed to Se^2−^ of MoSe_2_. The Raman spectra also confirmed the successful formation of MoSe_2_ on the surface of Mo electrode as shown in Figure S17 (Supporting Information). Combining with the electrodes with or without MoSe_2_, the band alignment of Sb_2_S_3_ planar solar cells is then sketched in Figure 5d, in which the band energy level data are referred from the literatures.^[^ ^11^, ^28^ ^]^ Compared with Mo electrode without MoSe_2_, the VBM of Mo electrode with MoSe_2_ is found to be closer to the VBM of Sb_2_S_3_ which observably reduces the hole extraction barrier, as well as nonradiative recombination at the Sb_2_S_3_/Mo interface. Thus, such a cascade band alignment delivers a lower contact resistance at back interface and promotion of hole collection efficiency. It is noticed that although the introduction of MoSe_2_ ca be facilely realized by pre‐salinization of Mo bottom electrode at high temperature and long duration (i.e., 550 °C for 30 min proposed by Deng et al.)^[^ ^11^ ^]^ in the substrate‐based Sb_2_S_3_ solar cells, the Se‐modification of Mo top electrode is still full of challenges due to low melting point of Sb_2_S_3_. Hence the pre‐selenization of Mo target developed in this work provides a potential regulation path for Mo/Sb_2_S_3_ interface.

To assess the impact of band alignment engineering, the device performances are tested by the *J–V* in Sb_2_S_3_ devices with MoSe_2_ buffer layer as shown in Figure 5e. Notably, the roll‐over effect completely disappears at the high voltage region, indicating the modified back contact electrodes remarkably reduce the hole collection barrier after the introduction of MoSe_2_ layer. And the device parameters of the representative devices are shown in Table S4 (Supporting Information), indicating a remarkable improvement of PCE from 4.2% to 5.1%, delivering a *V*_oc_ of 0.709 V, a *J*_sc_ of 14.7 mA·cm^−2^, and *FF* of 48.6%. Typically, the device displays a negligible hysteresis, confirmed by the almost same *J–V* curves measured under both reverse and forward scan directions (Figure S18, Supporting Information). Such facile engineering of the back contact interface leads to an obvious increase in *V*_oc_ and *FF* and consequentially boosts PCE. In order to demonstrate the superiority of the MoSe_2_ layer, the EQE test result along with the integral curve corresponding to the champion device are also given in Figure 5f. It shows an absorption value up to 87.4% at 540 nm, which is higher than Sb_2_S_3_ solar cells without MoSe_2_, which manifests that the additional MoSe_2_ is conducive to carrier separation and transport and improves the overall efficiency of the device. To further illustrate it, the *J–V* test in the dark (Figure 5g) shows the lowest leakage current of 1.2E‐7 mA·cm^−2^ in the MoSe_2_ equipped device, indicating a decrease in leakage current compared with that without MoSe_2_. Generally, the leakage current here is the result of the Shockley–Read–Hall recombination of carriers in the depletion layer or interface.^[^ ^7^ ^]^ As the fabrication procedure of CdS/Sb_2_S_3_ junction is identical, the relatively smallest leakage current of the MoSe_2_ modified device can be ascribed to the formation of benign back contact interface which is the reason for the increase in *V*_oc_. Besides, the dependences of *V*_oc_ and *J*_sc_ on light intensity are also recorded to investigate the recombination kinetics of the solar cell. Figure 5h shows the *V*_oc_ measured as a function of the light intensity and their dependence is linearly fitted by the following Equation (1):^[^ ^30^ ^]^

(1) $$V\mathrm{oc}=n\frac{kT}{q}\ln\left(I\right)+C$$

where *I* is the light intensity, *q* refers to elementary charge, *T* is absolute temperature, *k* is the Boltzmann constant, *C* is a constant, and *n* is the ideal factor connected with recombination. According to the slope of the fitted line, the values of *n* are determined to be 2.184 and 1.425 for the Sb_2_S_3_ solar cells without and with MoSe_2_, respectively. The lower *n* of the MoSe_2_ layer with Sb_2_S_3_ device indicates the monomolecular trap‐assisted Shockley–Read–Hall (SRH) recombination at the back contact interface can be effectively inhibited. In addition, as depicted in Figure 5i, the function between *J*_sc_ and light intensity can be studied according to Equation (2):

(2) $$J\mathrm{sc}\propto I^{\alpha }$$

where *I* is the light intensity and *α* represents power‐law component.^[^ ^31^ ^]^ It can be concluded from the fitting line that the values of *α* are determined to be 0.8 and 0.97 for the pristine Sb_2_S_3_ and MoSe_2_‐basd Sb_2_S_3_ solar cells, respectively. This larger *α* value means more efficient carrier collection. In brief, the favorable modification of back contact interface by an additional MoSe_2_ layer significantly improve the performance of Mo‐based superstrate Sb_2_S_3_ solar cells.

Finally, the normalized PCE of the best device (unencapsulated) as a function of the expose time in ambient conditions with 45% humidity, ≈26 °C room temperature is shown in Figure S19 (Supporting Information). Specifically, the device still maintains 90.5% of the initial PCE after storing for 13 months without encapsulation, indicating superior device stability with Mo electrodes. Such a Mo electrodes, combining with the careful interface engineering, enables to achieve a low‐cost and full‐inorganic superstrate Sb_2_S_3_ solar cell.

## 3 Conclusion

In this work, we have successfully applied a low‐cost Mo electrode in superstrate structured Sb_2_S_3_ solar cells for the first time, breaking the limitation of only noble‐Au metal electrode. An additional Se layer is also skillfully used to softly load Mo particles, as well as an adhesive layer to form Sb_2_Se_3_ to improve the device back contact due to the deep VBM of Sb_2_S_3_ surface. To further overcome the roll‐over issue, we artfully design a pre‐selenized Mo target and then incorporate it into the device. Finally, the device with a MoSe_2_‐modified Mo electrode has achieved an impressive PCE of 5.1%, which is among the highest PCE of Sb_2_S_3_ solar cells equipped with metal electrode except for Au. This research provides a potential low‐cost Mo electrode for superstrate structured Sb_2_S_3_ solar cells, also reveals a universal interface engineering strategy for other low‐cost and highly stable electrode deposition.

## 4 Experimental Section

### Chemical

Cadmium nitrate tetrahydrate (Cd(NO_3_)_2_·4H_2_O, AR, Sinopharm), ammonium hydroxide (NH_3_·H_2_O, 25%–28%, Sinopharm), thiourea (CH_4_N_2_S, AR, Sinopharm), cadmium chloride hemipentahydrate (CdCl_2_·2.5H_2_O, AR, Sinopharm), methanol (CH_3_OH, AR, Sinopharm), sodium thiosulfate pentahydrate (Na_2_S_2_O_3_·5H_2_O, AR, Sinopharm), potassium antimony tartrate (C_8_H_4_K_2_O_12_Sb_2_·3H_2_O, AR, Macklin), and selenium (Se, 99.999%, Aladdin), all the chemicals were used as received without further purification.

### Fabrication of *P–N* Junction

The *P–N* junction structure of the Sb_2_S_3_ planar solar cell was FTO/CdS/Sb_2_S_3_. To build a *P–N* junction of CdS/Sb_2_S_3_, the FTO conductive glass as device substrate was ultrasonically cleaned with detergent, acetone, anhydrous ethanol, and deionized water in sequence for 1 h. After drying, the substrate was treated by UV ozone cleaner for 20 min. The CdS layer was then deposited using chemical bath deposition method carried out at 65 °C for 15 min in a solution of 100 ml of deionized water, 0.15 mmol of Cd(NO_3_)_2_·4H_2_O, 9.4 mmol of CH_4_N_2_S, and 0.19 mol of NH_3_·H_2_O. To improve the crystallinity, the as‐prepared CdS was further treated with CdCl_2_. In this process, as‐prepared CdS was spin‐coated with CdCl_2_ (40 µl, 0.05 g CdCl_2_ is soluble in 10 mL methanol) at 2500 rpm for 30 s. Subsequently, the CdS substrate was annealed in air at 380 °C for 5 min. Afterward, the hydrothermal method was adopted for the deposition of Sb_2_S_3_ as an absorber layer according to the previously reported recipe. The Sb_2_S_3_ film was synthesized by using Na_2_S_2_O_3_·5H_2_O and C_8_H_4_K_2_O_12_Sb_2_·3H_2_O, and selenourea as S and Sb sources, respectively. In brief, 30 mm C_8_H_4_K_2_O_12_Sb_2_·3H_2_O and 60 mm Na_2_S_2_O_3_·5H_2_O were added into a Teflon tank (100 ml) of an autoclave that contained 80 ml of ultrapure water, which was then heated at 120 °C for 12 h. After the hydrothermal reaction, the sample was washed with ultrapure water and dried on a hotplate at 70 °C for 10 min under atmospheric pressure. Finally, the unannealed *P–N* junction CdS/Sb_2_S_3_ was obtained.

### Solar Cells Fabrication

The FTO/CdS/Sb_2_S_3_/Mo structure, Mo was deposited directly on the as‐prepared FTO/CdS/Sb_2_S_3_ (before and after annealing) by magnetron sputtering (150 °C, 100 W, 2 h) as a back electrode, which could be seen in Figure 1. Finally, the unannealed samples were further annealed at 350 °C for 10 min in a nitrogen atmosphere to improve its quality. To insert a Se functional layer, Se (0.01, 0.1, and 0.3 g) were evaporated on Sb_2_S_3_ surface before Mo sputtering. The evaporation conditions were 1 Pa pressure, 10 cm of source‐substrate distance, and complete evaporation of powder source for 15 s under 120 A of current. As for MoSe_2_ layer, the Mo target was first treated with Se (0.005 g) vaporization (consistent with the above Se evaporation conditions), and then annealed on the hot table under N_2_ atmosphere at 500 °C for 20 min. Sequentially, the pre‐selenzied Mo was sputtered under the same procedure with the pristine Mo sputtering.

### Devices Characterizations

The crystal structure of Sb_2_S_3_ film was characterized by X‐ray diffraction (XRD, D/Max‐rA). The microstructure was observed using high‐resolution field emission scanning electron microscopy (FE‐SEM). The surface composition was characterized by X‐ray photoelectron spectroscopy (XPS, Shimadu, AXIS SUPRA+). The *J–V* curves of the solar cells were characterized using a Keithley 4200‐SCS semiconductor characterization system equipped with an AAASAN‐EI ELECTRIC solar simulator (XES‐40S1). The solar simulator illumination intensity was calibrated by a monocrystalline silicon reference cell (PV Measurements, PVM959) calibrated by the National Renewable Energy Laboratory (NREL). The square resistance of the Mo was observed using four probe test (RTS‐8). The EQE of the devices was performed by a solar cell quantum efficiency measurement system (Model QEX10).

## Conflict of Interest

The authors declare no conflict of interest.

## Supporting information

Supporting Information

## Data Availability

The data that support the findings of this study are available from the corresponding author upon reasonable request.
