# Supplementary material for: Sputtering of Molybdenum as a Promising Back Electrode Candidate for Superstrate Structured Sb2S3 Solar Cells
Source: Adv Sci (Weinh). 2023 Sep 5;10(30):2303414. doi: 10.1002/advs.202303414 (PMC10602520; doi:10.1002/advs.202303414)
Supplement: Supplementary file 1 — Supporting Information [file ADVS-10-2303414-s001.pdf]

ADVANCED SCIENCE
Open Access

Supporting Information

for *Adv. Sci.*, DOI 10.1002/advs.202303414

Sputtering of Molybdenum as a Promising Back Electrode Candidate for Superstrate Structured Sb_2S_3 Solar Cells

*Hu Li, Guo-Qin Yang, Xiao-Yang Hu, Yi-Hua Hu, Rui-Bo Zeng, Jin-Rui Cai, Li-Quan Yao, Li-Mei Lin, Li-Ping Cai and Guilin Chen**

Sputtering of Molybdenum as a Promising Back Electrode Candidate for Superstrate Structured Sb_2S_3 Solar Cells

Hu Li[a], Guo-Qin Yang[b], Xiao-Yang Hu[a], Yi-Hua Hu[a], Rui-Bo Zeng[a], Jin-Rui Cai[a], Li-Quan Yao[a], Li-Mei Lin[a], Li-Ping Cai[c], and Guilin Chen[a,*]

a. Fujian Provincial Engineering Technology Research Center of Solar Energy Conversion and Energy Storage, College of Physics and Energy, Fujian Normal University, Fuzhou, 350117, China.

b. State Grid Dehua County Electric Power Supply Company, Quanzhou, 362500, China.

c. College of Computer and Cyber Security, Fuzhou, 350117, China.

*Corresponding authors: Gui-Lin Chen (Prof. Chen)

E-mail: glchen@fjnu.edu.cn (G.L. Chen)

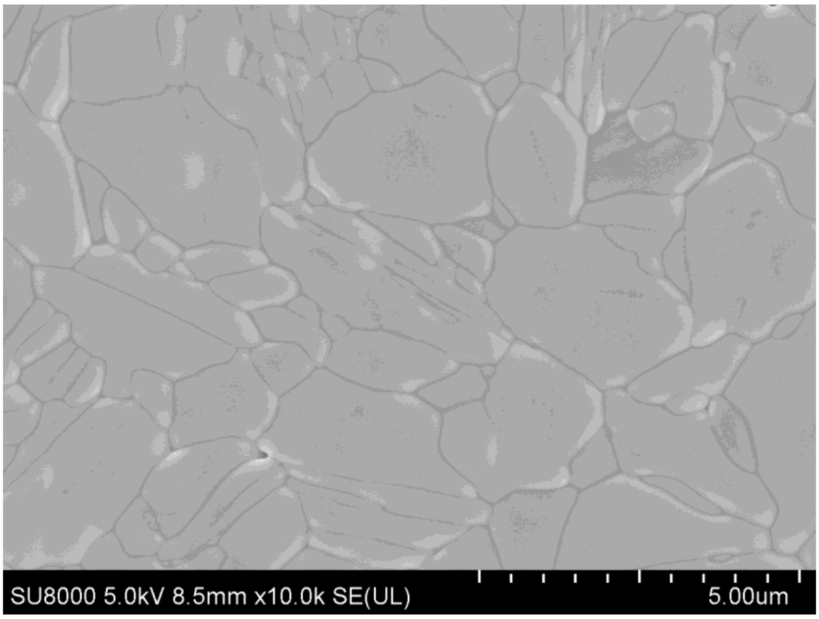

Figure S1 The SEM image of as-annealed Sb_2S_3 thin film.

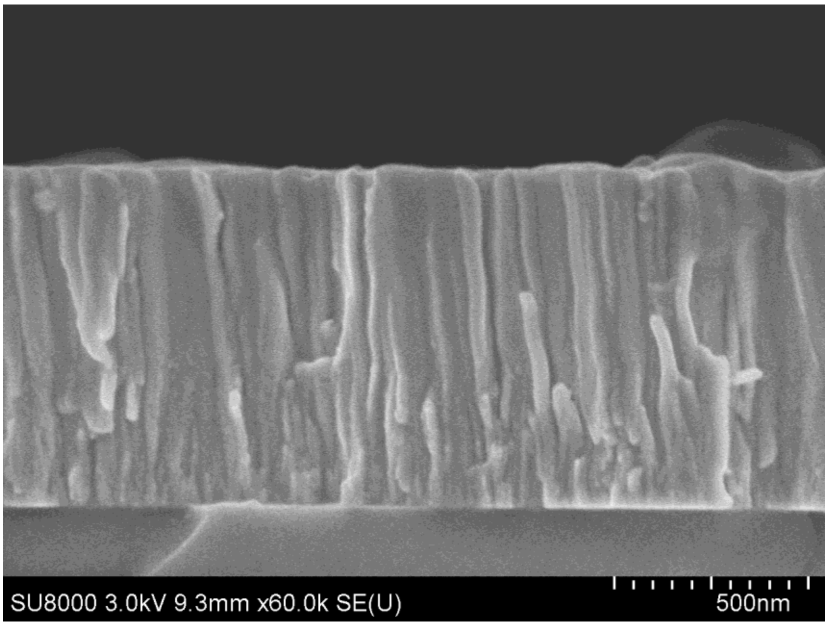

Figure S2 Cross-sectional SEM of Mo deposited on the glass substrate.

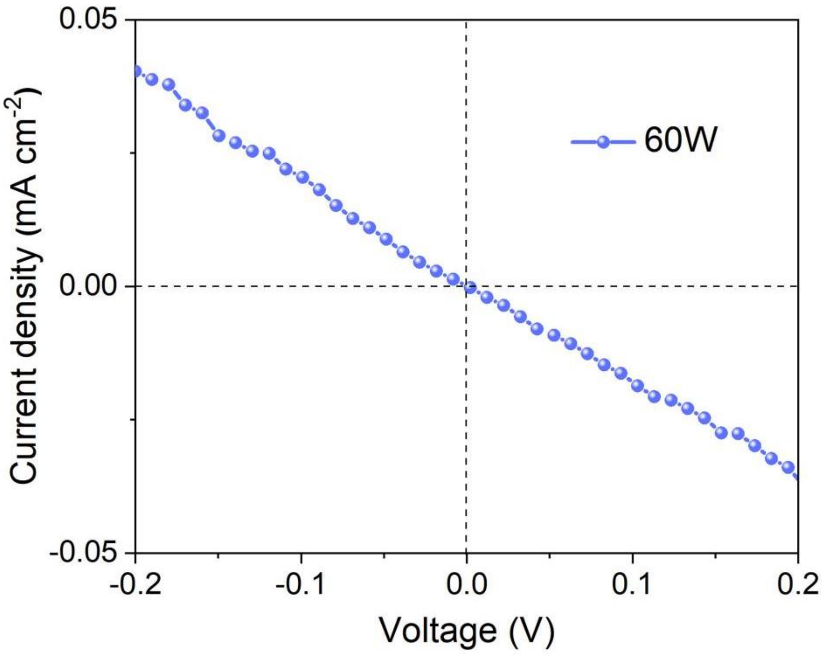

Figure S3 The *J-V* curve of the Sb_2S_3 solar cell with Mo electrode sputtered at 60 W.

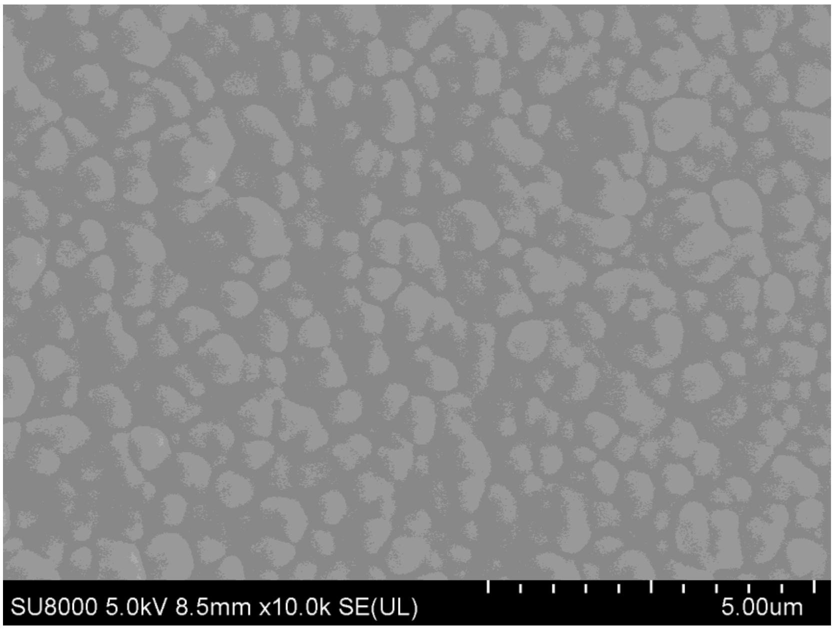

Figure S4 The SEM image of unannealed Sb_2S_3 film.

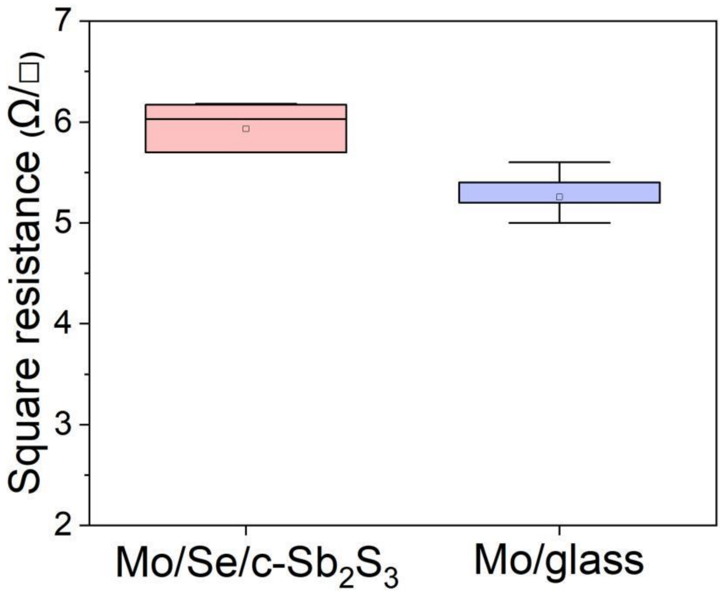

Figure S5 Square resistance of Mo deposited on Se/Sb_2S_3 layer or glass substrate.

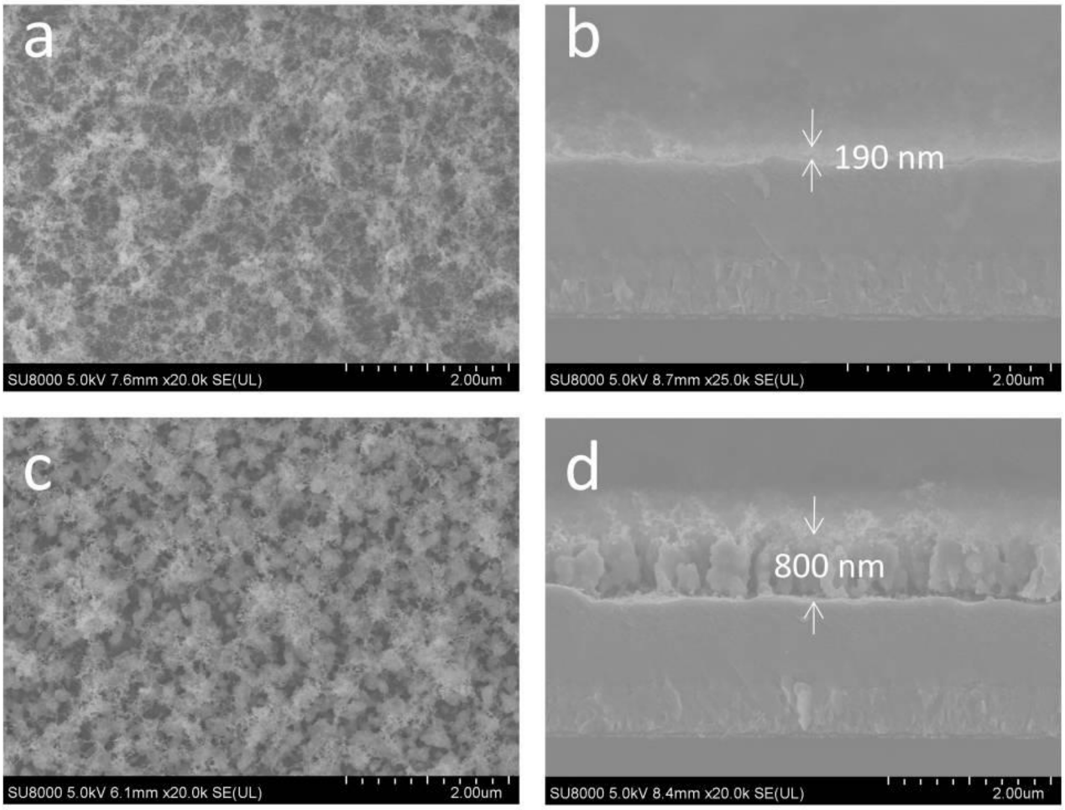

Figure S6 The surface and cross-sectional images of (a, b) 0.01g, and (c, d) 0.3g Se evaporated on Sb_2S_3 thin films.

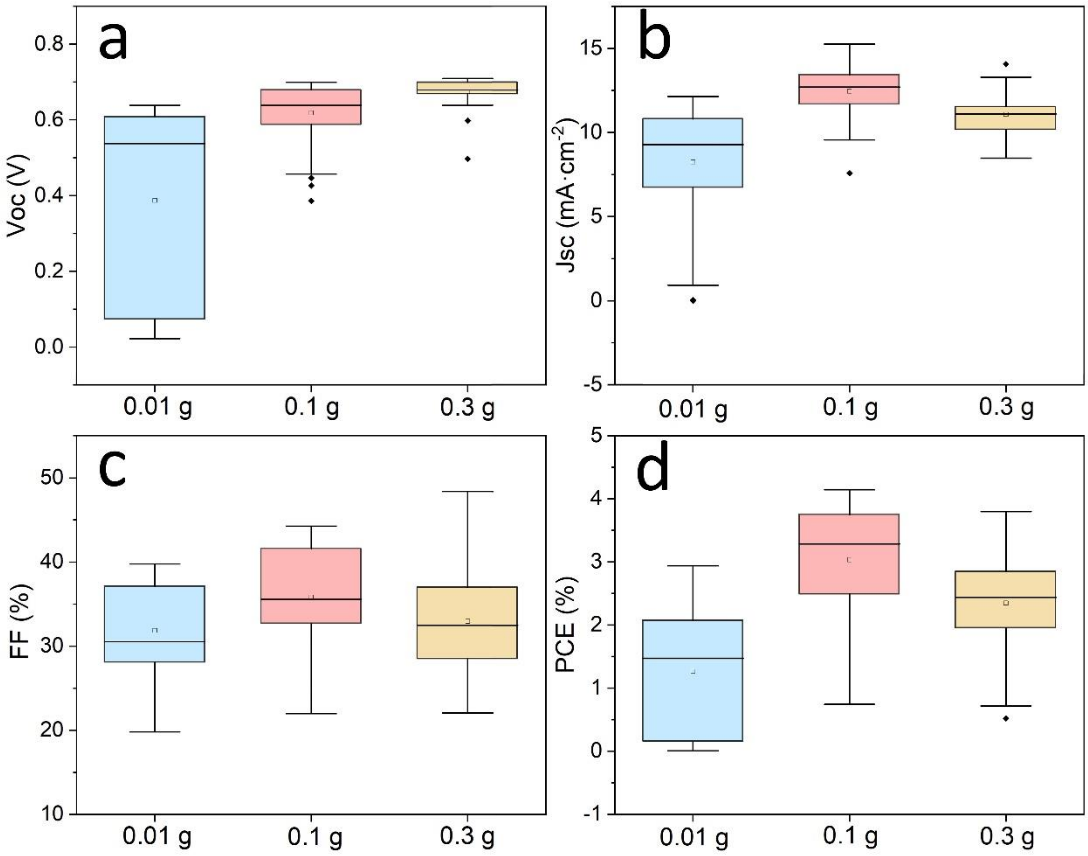

Figure S7 The data statistics charts about (a) open-circuit voltage, (b) current density, (c) fill fact, and (d) efficiency of the Sb_2S_3 solar cells with different Se layers.

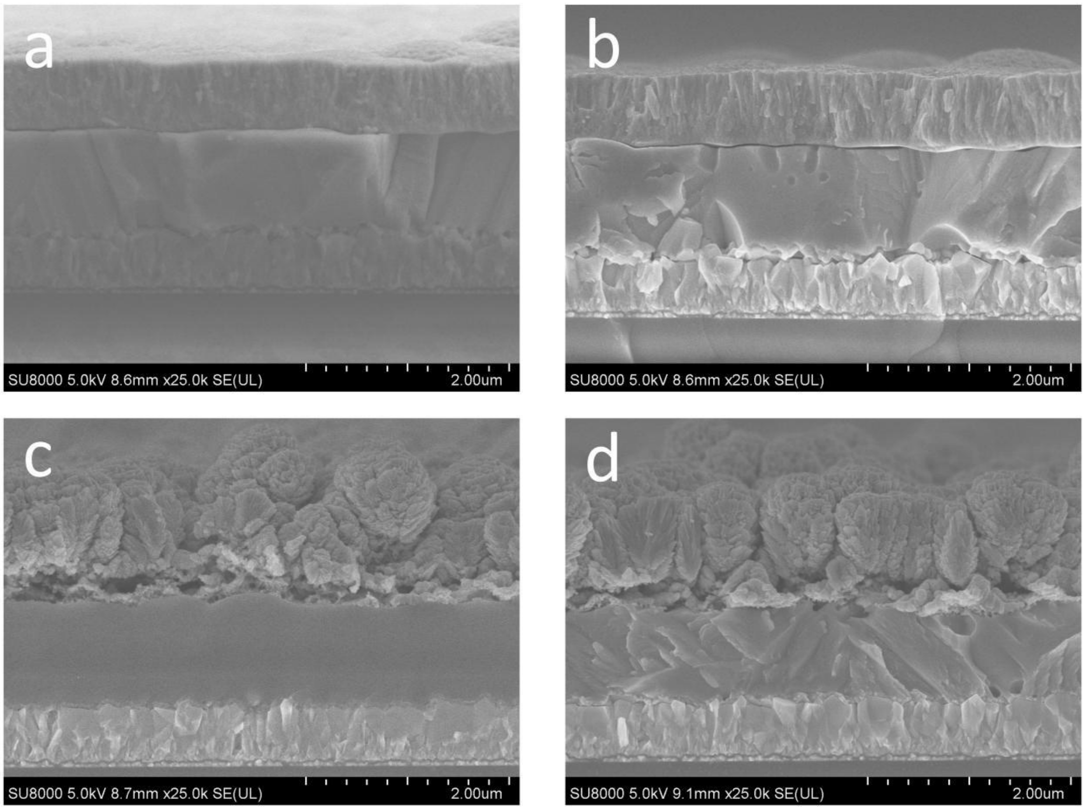

Figure S8 The cross-sectional images of (a, c) before- and (b, d) after-annealing Sb_2S_3 thin films with (a, b) 0.01g and (c, d) 0.3g Se layer.

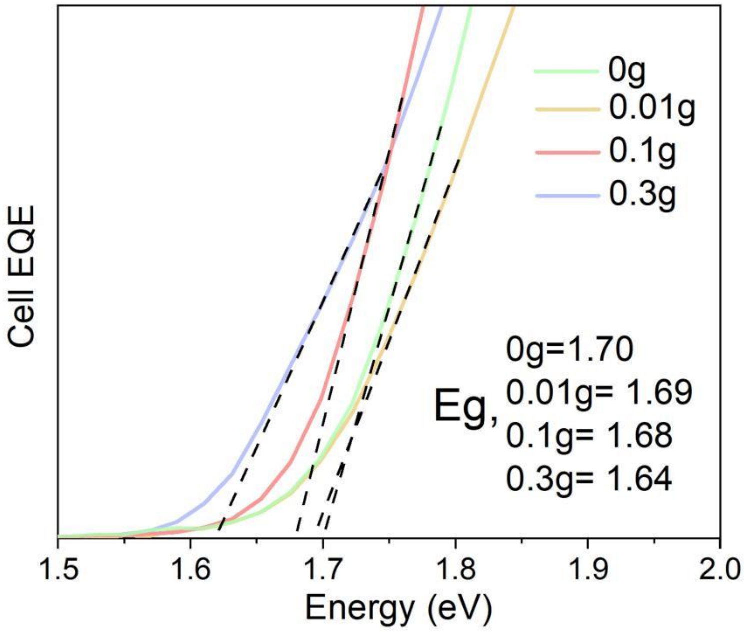

Figure S9 Bandgap calculation of the Sb_2S_3 film treated with 0g, 0.01g, 0.1g, and 0.3g Se layer.

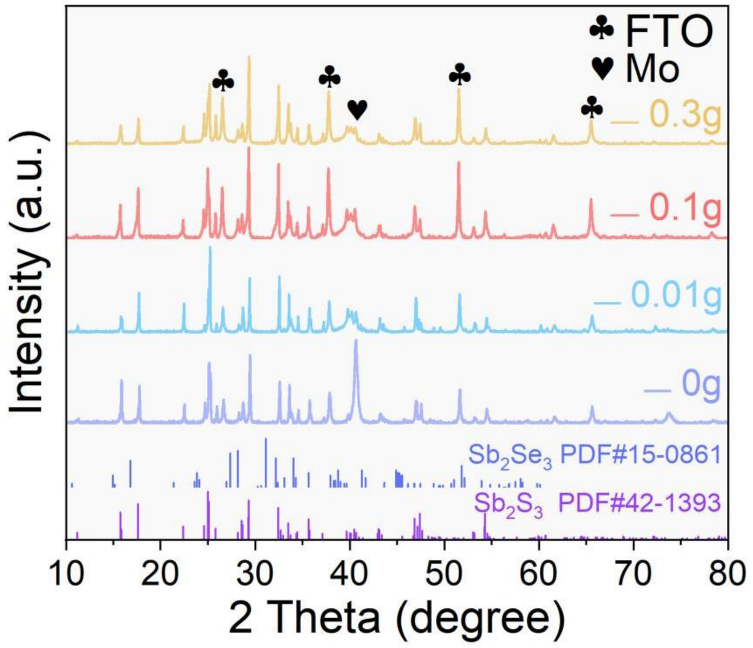

Figure S10 XRD patterns of the Sb_2S_3 solar cell modified with 0.01g, 0.1g, and 0.3g Se-layer.

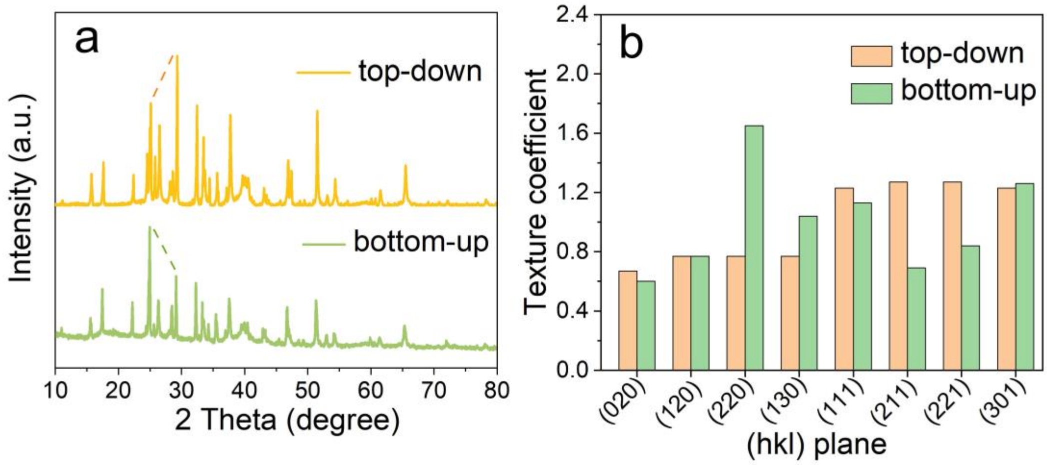

Figure S11 (a) XRD patterns of the Sb_2S_3 solar cells with different heat conduction direction. (b) The texture coefficients (TC) about different growth models deduced from XRD patterns.

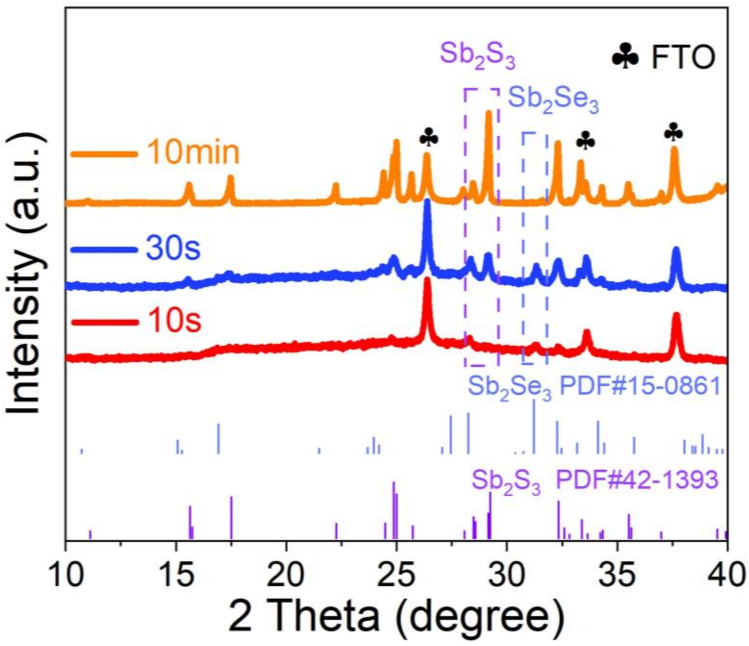

Figure S12 XRD patterns of the Sb_2S_3 solar cells with different heat treatment times.

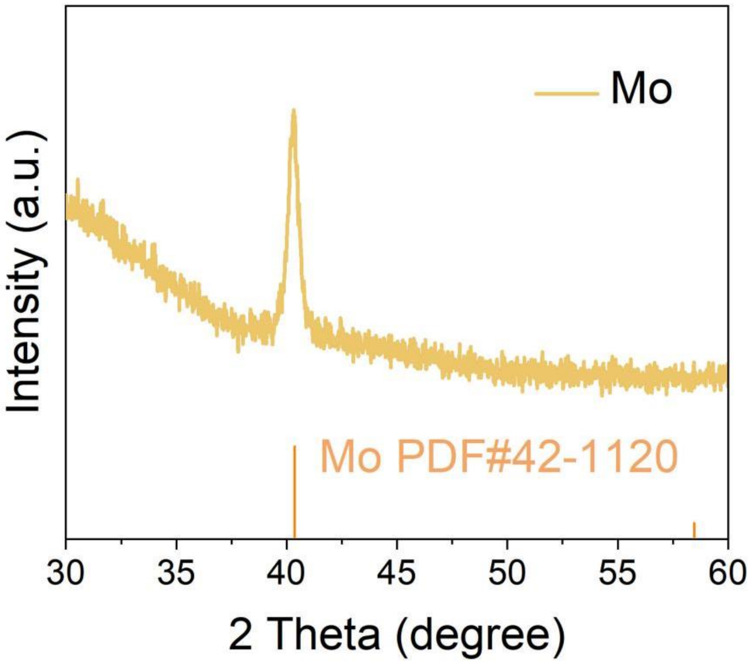

Figure S13 XRD pattern of the Mo film annealed under Se atmosphere at 350 ℃ for 10 min.

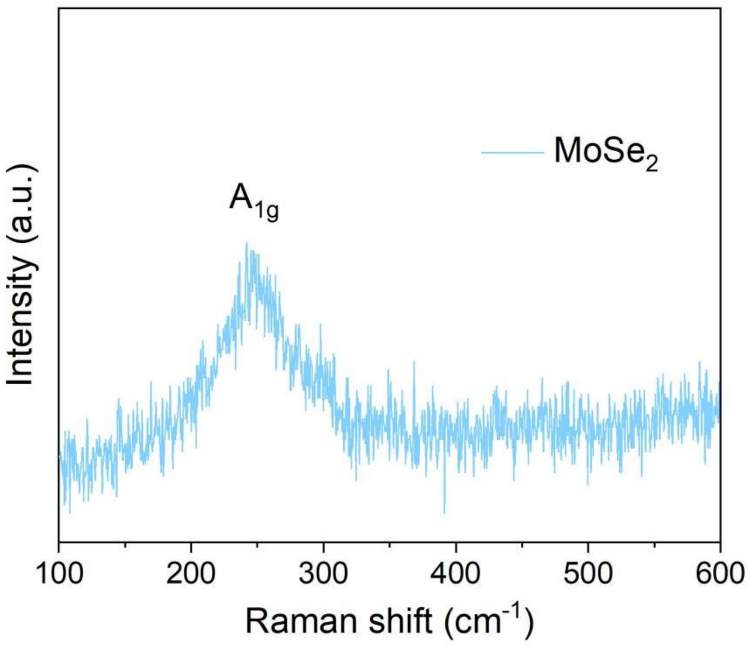

Figure S14 Raman spectra of Mo film sputtered with pre-selenized Mo target on glass for 1 minute.

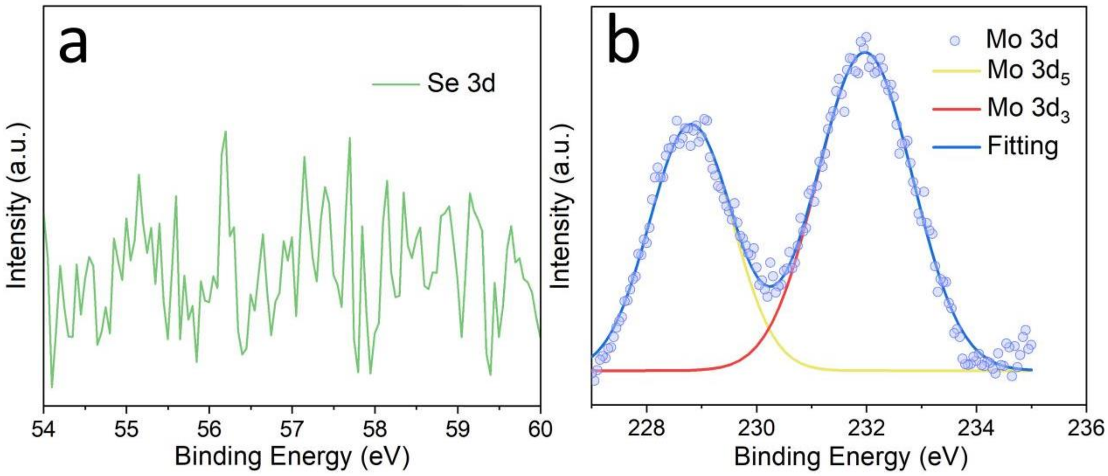

Figure S15 The XPS curves of Mo film sputtered with pre-selenized Mo target on glass for 10 minutes, (a) Se 3d and (b) Mo 3d.

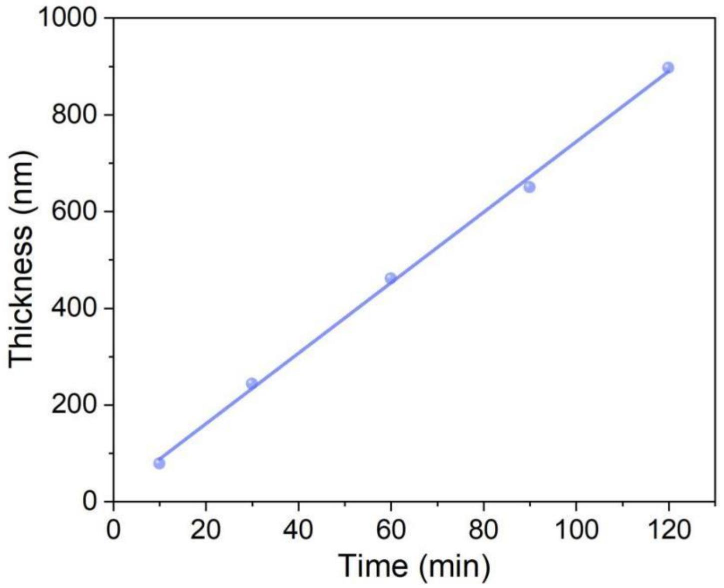

Figure S16 Mo electrode thickness measured from cross-sectional SEM changes as a function of sputtering time.

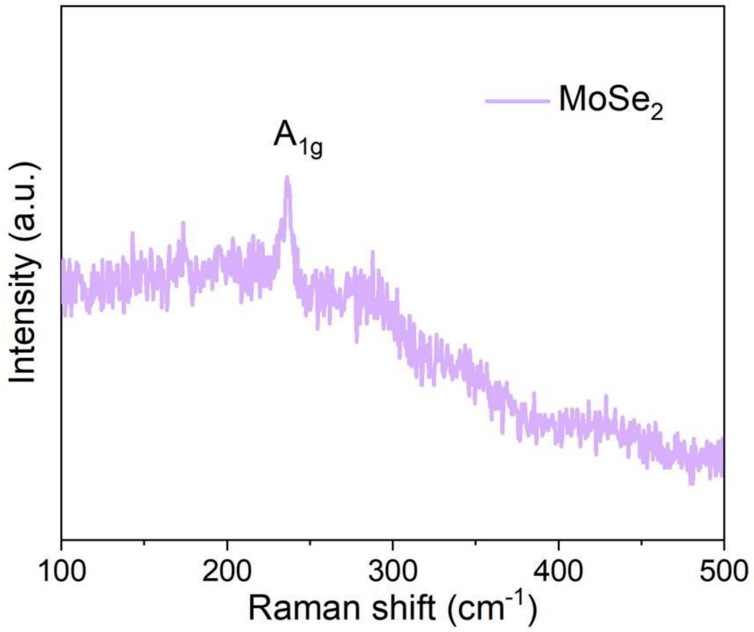

Figure S17 Raman spectra of $MoSe_2$ on Mo electrode peeled by tape.

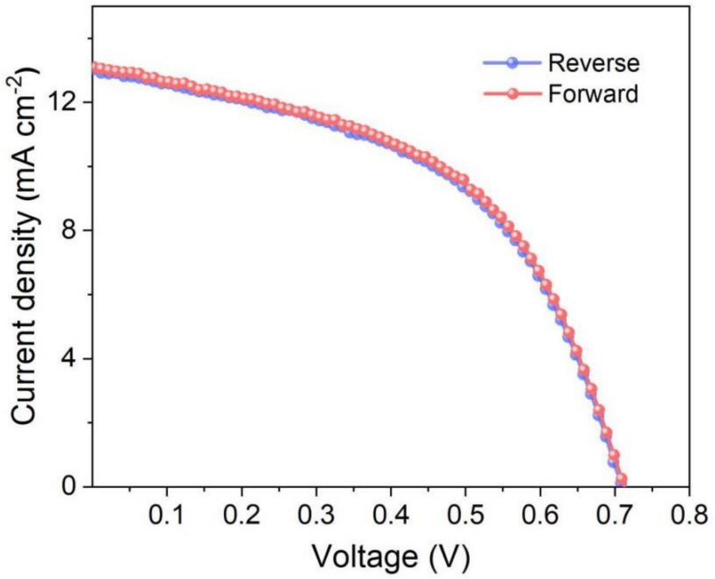

Figure S18 *J*-*V* hysteresis behavior of the Sb_2S_3 solar cell.

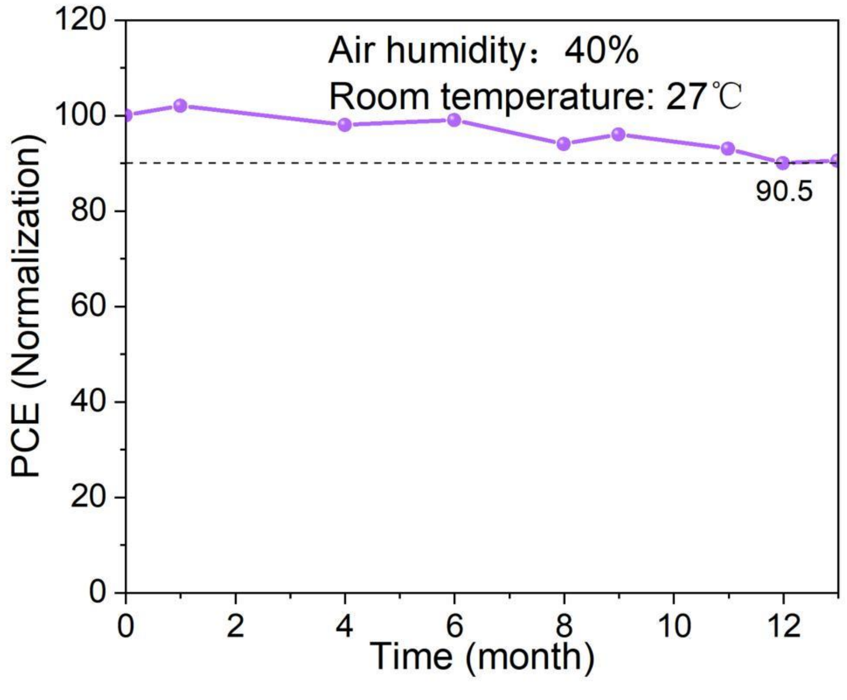

Figure S19 The relationship of PCE and exposes time.

Table S1 Photovoltaic performances of Sb_2S_3 solar cells based on organic HTL and Au electrode.

Device Structure	*Voc* [V]	Jsc [mA·cm^{-2}]	*FF* [%]	PCE [%]	Year/Ref
TiO_2/Sb_2S_3/P3HT/PEDOT:PSS/Au	0.51	14.2	54	3.9	2013[S1]
TiO_2/Sb_2S_3/PCPDTBT:PC_{60}BM/Au	0.59	16.1	66.5	6.4	2015[S2]
TiO_2/Sb_2S_3/P3HT/Au	0.59	11.71	56.89	3.97	2019[S3]
CdS/Sb_2S_3/Spiro-OMeTAD/Au	0.757	17.41	60.48	8.0	2022[S4]

Table S2 Photovoltaic performances of Sb_2S_3 solar cells with Mo electrode.

Device Structure	*Voc* [V]	*Jsc* [mA·cm^{-2}]	*FF* [%]	PCE [%]	Year/Ref
Mo/Sb_2S_3/CdS/ITO/Ag	0.310	10.53	31.29	0.99	2020[S5]
Mo/Sb_2S_3/CdS/i-ZnO/AZO/Al	0.506	5.73	33.70	0.98	2022[S6]
Mo/Sb_2S_3/CdS/i-ZnO/AZO/Al	0.191	9.70	35.00	0.65	2017[S7]
Mo/Sb_2S_3/CdS/i-ZnO/AZO/Ni:Al	0.628	14.10	37.80	3.35	2023[S8]
Mo/Sb_2S_3/CdS/i-ZO/AZO/Ni:Al	0.574	5.96	37.78	1.29	2019[S9]
Mo/$MoSe_2$/Sb_2S_3/CdS/i-ZO/AZO/Ni:Al	0.466	13.60	31.06	1.86	2022[S10]
Mo/$MoSe_2$/Sb_2S_3/CdS/ITO/Ag	0.654	11.60	49.50	3.75	2023[S11]

Table S3 Sb_2S_3 device parameters with various Se layers.

	Voc [V]	*Jsc* [mA·cm^{-2}]	*FF* [%]	PCE [%]
0.01g Se	0.578	9.9	36.2	2.1
0.1g Se	0.699	13.6	44.1	4.2
0.3g Se	0.679	11.3	37.3	2.9

Table S4 Champion device parameters.

	Voc [V]	Jsc [mA·cm^{-2}]	FF [%]	PCE [%]
Control	0.699	13.6	44.1	4.2
Pre-selenization	0.709	14.7	48.6	5.1

References

[S1] T. Fukumoto, T. Moehl, Y. Niwa, M. K. Nazeeruddin, M. Cratzel, L. Etgar, Adv. Energy Mater. **2013**, 3, 29-33.

[S2] Y. C. Choi, S. I. Seok, Adv. Funct. Mater. **2015**, 25, 2892-2898.

[S3] W. H. Kim, S. Woo, K. P. Kim, S. M. Kwon, D. H. Kim, Nanoscale Res. Lett. **2019**, 14:25.

[S4] S. Wang, Y. Zhao, B. Che, C. Li, X. Chen, R. Tang, J. Gong, X. Wang, G. Chen, T. Chen, J. Li, X. Xiao, Adv. Mater. **2022**, 2206242.

[S5] J. Luo, W. Xiong, G. Liang, Y. Liu, H. Yang, Z. Zheng, X. Zhang, P. Fan, S. Chen, J. Alloys Compd. **2020**, 826, 154235.

[S6] P. S. Pawar, R. Nandi, K. E. Neerugatti, J. Y. Cho, J. Heo, J. Alloys Compd. **2022**, 898, 162891.

[S7] L. Zhang, D. Zhuang, M. Zhao, Q. Gong, L. Guo, L. Ouyang, R. Sun, Y. Wei, X. Lyu, X. Peng, Mater. Lett. **2017**, 208, 58-61.

[S8] Z. Peng, Q. Zheng, R. Wang, L. Sun, H. Wang, Y. Yuan, Y. Xing, L. Yao, J. Bi, W. Li, Sol. Energy Mater. Sol. Cells **2023**, 253, 112208.

[S9] G. Pan, D. Wang, S. Gao, P. Gao, Q. Sun, X. Liu, Z. Zhou, Y. Sun, Y. Zhang, Sol. Energy **2019**, 182, 182, 64-71.

[S10] L. Liu, S. L. Zhang, J. Y. Wu, W. H. Wang, W. Liu, L. Wu, Y. Zhang, Chin. Phys. B **2020**, 29, 058801.

[S11] H. Deng, Y. Cheng, Z. Chen, X. Lin, J. Wu, Q. Zheng, C. Zhang, S. Cheng, Adv. Funct. Mater. **2023**, 33, 2212627.
